# Combined cardiac, lung, and diaphragm ultrasound for predicting weaning failure during spontaneous breathing trial

**DOI:** 10.1186/s13613-024-01294-2

**Published:** 2024-04-20

**Authors:** Jia Song, Qiancheng Luo, Xinle Lai, Weihang Hu, Yihua Yu, Minjia Wang, Kai Yang, Gongze Chen, Wenwei Chen, Qian Li, Caibao Hu, Shijin Gong

**Affiliations:** 1 Department of Critical Care Medicine, Zhejiang Hospital, No. 12, Lingyin Road, Xihu District, Hangzhou, Zhejiang 310013 China; 2 Department of Critical Care Medicine, Shanghai Pudong New Area Gongli Hospital, No. 219, Miaopu Road, Pudong New Area, Shanghai, 200135 China; 3 The 2nd Clinical Medical College, Zhejiang Chinese Medical University, No. 548, Binwen Road, Binjiang District, Hangzhou, 310053 China

**Keywords:** Weaning, Mechanical ventilation, Spontaneous breathing trial, Echocardiography, Lung ultrasound, Diaphragm ultrasound

## Abstract

**Background:**

Weaning from invasive mechanical ventilation (MV) is a complex and challenging process that involves multiple pathophysiological mechanisms. A combined ultrasound evaluation of the heart, lungs, and diaphragm during the weaning phase can help to identify risk factors and underlying mechanisms for weaning failure. This study aimed to investigate the accuracy of lung ultrasound (LUS), transthoracic echocardiography (TTE), and diaphragm ultrasound for predicting weaning failure in critically ill patients.

**Methods:**

Patients undergoing invasive MV for > 48 h and who were readied for their first spontaneous breathing trial (SBT) were studied. Patients were scheduled for a 2-h SBT using low-level pressure support ventilation. LUS and TTE were performed prospectively before and 30 min after starting the SBT, and diaphragm ultrasound was only performed 30 min after starting the SBT. Weaning failure was defined as failure of SBT, re-intubation, or non-invasive ventilation within 48 h.

**Results:**

Fifty-one patients were included, of whom 15 experienced weaning failure. During the SBT, the global, anterior, and antero-lateral LUS scores were higher in the failed group than in the successful group. Receiver operating characteristic curve analysis showed that the areas under the curves for diaphragm thickening fraction (DTF) and global and antero-lateral LUS scores during the SBT to predict weaning failure were 0.678, 0.719, and 0.721, respectively. There was no correlation between the LUS scores and the average E/e’ ratio during the SBT. Multivariate analysis identified antero-lateral LUS score > 7 and DTF < 31% during the SBT as independent predictors of weaning failure.

**Conclusion:**

LUS and diaphragm ultrasound can help to predict weaning failure in patients undergoing an SBT with low-level pressure support. An antero-lateral LUS score > 7 and DTF < 31% during the SBT were associated with weaning failure.

**Graphical Abstract:**

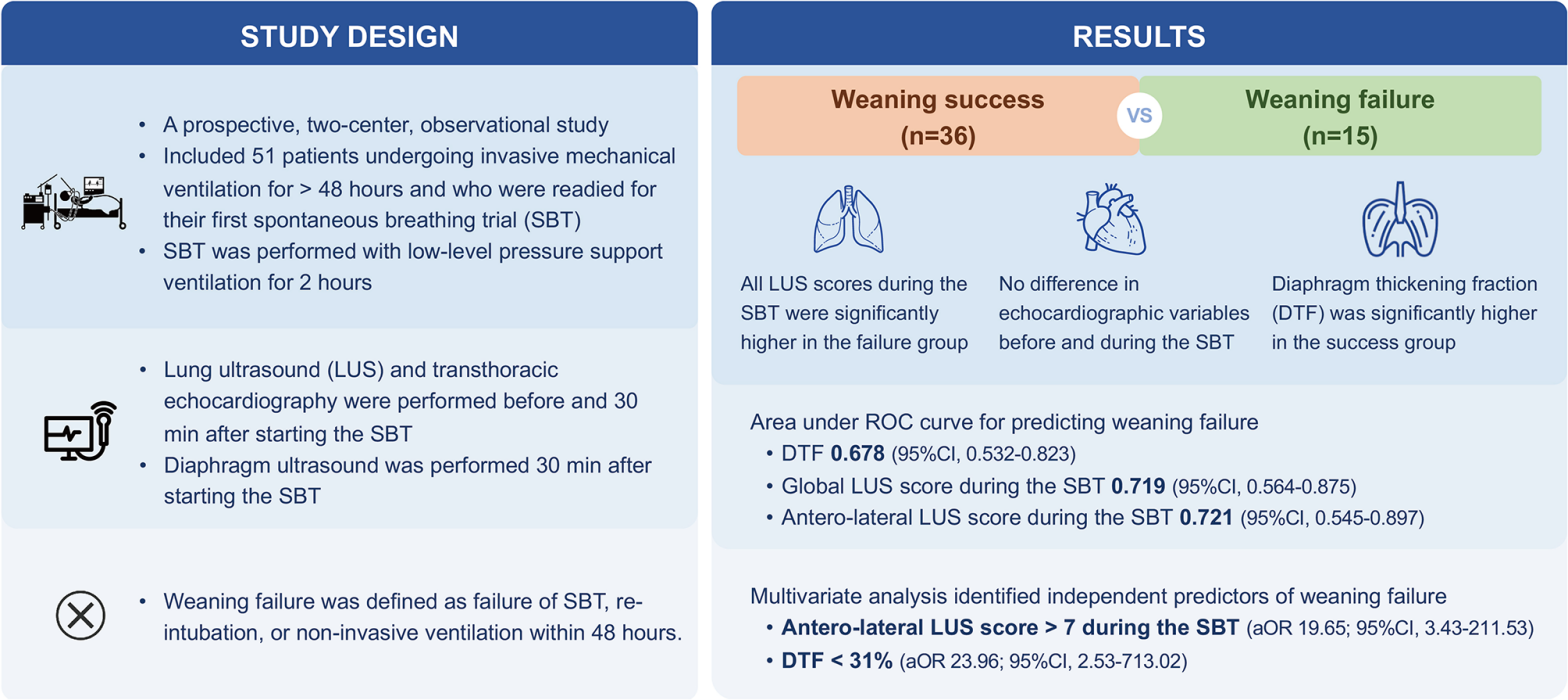

**Supplementary Information:**

The online version contains supplementary material available at 10.1186/s13613-024-01294-2.

## Background

Weaning from invasive mechanical ventilation (MV) is a major challenge in critically ill patients. In a large, prospective, international observational study, only 65% of intensive care unit (ICU) patients receiving more than 2 days of invasive ventilation were successfully weaned at day 90 [1]. Weaning failure is associated with prolonged ICU stay and increased risk of death [2, 3]. The transition from complete ventilatory support to spontaneous breathing is a complex process that places stress on multiple organs [4]. This process has the potential to unmask previously undetected organ dysfunctions, such as loss of lung aeration, ventilator-induced diaphragm dysfunction, and weaning-induced pulmonary edema (WIPO). Consequently, identifying the underlying mechanisms of weaning failure and implementing targeted interventions may thus have important prognostic implications.

Ultrasound is accepted as a reliable tool for monitoring physiological changes in the cardiorespiratory system during various stages of weaning [5–7]. Ultrasound assessment of heart, lung, and respiratory muscle functions has improved our understanding of the pathophysiological process of weaning and helped to predict weaning outcomes [8]. However, most published studies have primarily focused on single-organ assessments, overlooking the fact that weaning failure may depend on several factors and the intricate interplay between organs.

Hence, we assumed that a comprehensive ultrasound assessment of the heart, lungs, and diaphragm during the spontaneous breathing trial (SBT) process could help predict weaning outcome. This study aimed to investigate the accuracy of lung ultrasound (LUS), transthoracic echocardiography (TTE), and diaphragm ultrasound for predicting weaning failure and to determine which ultrasound parameters were associated with weaning failure in critically ill patients undergoing an SBT with low-level pressure support.

## Methods

### Patients

This prospective, observational study was conducted in two mixed intensive care units (ICUs) of tertiary hospitals in China over a 16-month period. This study was conducted according to the tenets of the Declaration of Helsinki. The protocol was approved by the ethics committee of our institution (approval number 2021–69 K). Written informed consent was obtained from each patient’s next of kin prior to participation.

Patients were eligible if they fulfilled the following inclusion criteria: (1) invasive MV for > 48 h; and (2) eligibility for their first SBT according to current guidelines (Electronic Supplementary Material 1) [9]. The exclusion criteria were as follows: (1) age < 18 years; (2) pregnancy; (3) presence of thoracostomy, pneumothorax, or pneumomediastinum; (4) presence of flail chest or rib fractures; (5) pre-existing cervical spinal injury, history or final diagnosis of neuromuscular disorders; (6) tracheostomy; (7) history or new detection of paralysis (no movement) or paradoxical movement of a single hemidiaphragm on diaphragmatic ultrasound; (8) atrial fibrillation, severe mitral valve disease, mitral valve replacement or repair; (9) poor quality of ultrasound images unsuitable for analysis; and (10) patient’s next of kin refused participation.

### Weaning trials

Eligible patients underwent an SBT with low-level pressure support (pressure-support level of 8 cmH_2_O and zero positive end-expiratory pressure) for 2 h, according to current guidelines [10, 11]. SBT was deemed to have failed and was terminated when one of the following signs occurred [9]: (1) acute respiratory distress (respiratory rate > 35 breaths/min); (2) SaO_2_ < 90% with an FiO_2_ ≥ 50%; (3) heart rate > 140 beats/min or an increase of ≥ 20%; (4) systolic arterial blood pressure ≥ 180 mmHg or an increase of ≥ 20%; and (5) change in mental status, agitation, or anxiety. If the patient successfully passed the SBT, the physicians in charge decided whether to extubate, independent of the investigators. In the event of SBT failure, the pre-SBT MV settings were resumed.

**Fig. 1:**
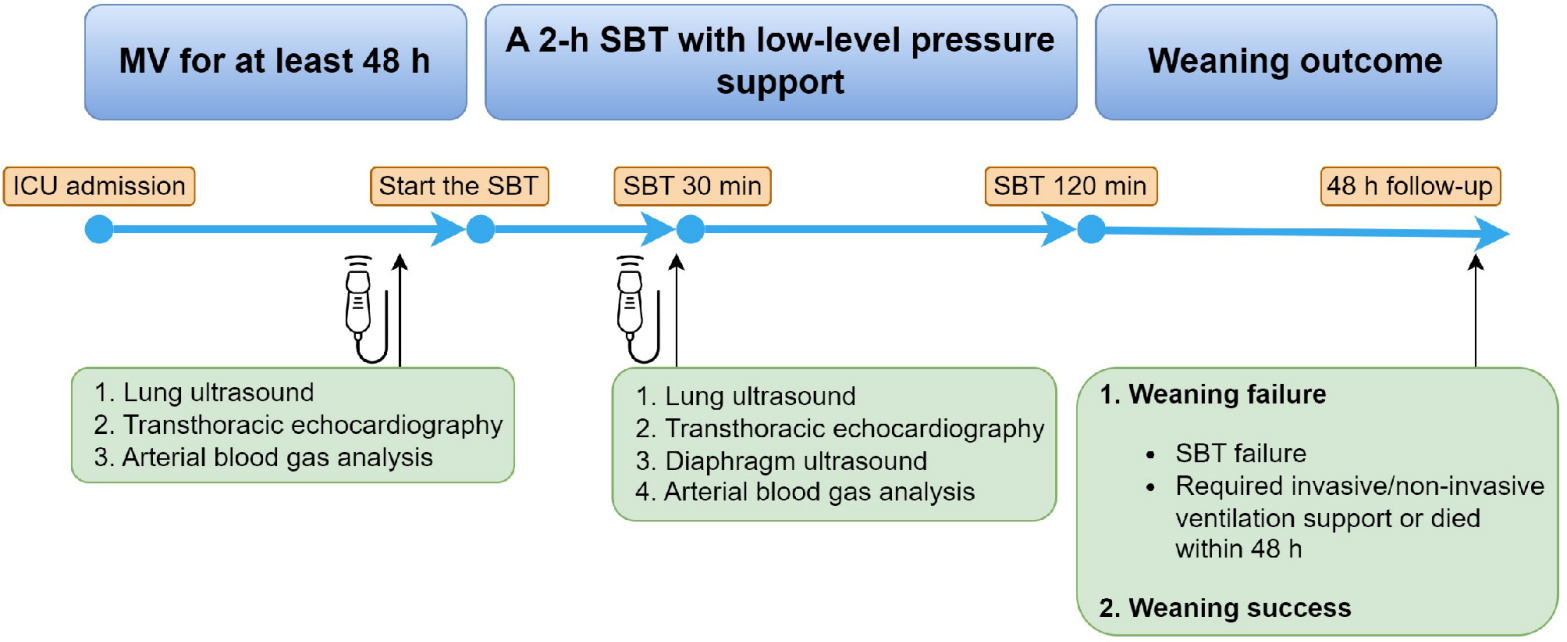
Timeline of study protocol. Patients who underwent invasive mechanical ventilation (MV) for > 48 h and who were eligible for their first spontaneous breathing trial (SBT) were included. An integrated ultrasound assessment of the heart, lungs, and diaphragm was performed before and 30 min after starting a 2-h SBT with low-level pressure support. After successfully passing the SBT, the physicians in charge decided whether to extubate, independent of the investigators. Weaning outcome was monitored for 48 h. Weaning failure was defined as failure of SBT, reintubation, or non-invasive ventilation within the following 48 h

Weaning failure was defined as either a failed SBT, or need for MV (invasive or non-invasive) within 48 h after extubation. The timeline of the study protocol is described in Fig. 1.

### Measurements and data collection

Upon inclusion, the following parameters were recorded: demographic data, comorbidities, reasons for ICU admission, Acute Physiology and Chronic Health Evaluation (APACHE) II score, Sequential Organ Failure Assessment (SOFA) score, albumin, cumulative fluid balance over last 3 days (2 days in case of MV < 3 days before the first SBT), use of diuretics before the SBT, duration of invasive MV, and ventilation parameters. Heart rate, blood pressure, respiratory rate, pulsed oxygen saturation, tidal volume, and blood gas analysis variables were also recorded before and 30 min after starting the SBT.

### Ultrasound examinations

All ultrasound examinations were performed using a CX-50 (Philips Healthcare, Amsterdam, The Netherlands) or Venue Go™ (GE Healthcare, Wuxi City, China) ultrasound device. TTE and LUS were performed before and 30 min after starting the SBT, and diaphragm ultrasound was only performed 30 min after starting the SBT. All examinations were performed by two fully trained and experienced operators (XLL and QCL) and reviewed off-line by an independent operator who was blinded to the medical charts (JS). Before this study, three investigators underwent standardized training sessions in cardiac, lung, and diaphragm ultrasound provided by the Chinese Critical Ultrasound Study Group (CCUSG) and obtained qualification certificates.

The techniques and parameters for conducting TTE, LUS, and diaphragm ultrasound evaluations are described in Electronic Supplementary Material 1.

### Statistical analysis

The sample size was calculated by considering an area under the receiver operating characteristic (ROC) curve > 0.80 as acceptable diagnostic accuracy. According to previous study [12], a weaning failure rate of 25% was assumed. We planned to include 48 patients, with a Type I error of 0.05 and a Type II error of 0.10 (power is 90%). Given an expected dropout rate of approximately 10% due to technical problems, we estimated that the sample size of 52 patients. The results are expressed as the median (interquartile range) for quantitative variables and the number and percentage for categorical variables. The patients were divided into weaning success and weaning failure groups according to the weaning outcome. Numerical data were compared between groups using a Mann–Whitney U test, and categorical data were compared using χ^2^ or Fisher’s exact tests. Data before and 30 min after starting the SBT were compared using a paired-samples Wilcoxon’s test. ROC curves were constructed to evaluate the performance of nine indices to predict weaning failure: global LUS score, anterior LUS score, antero-lateral LUS score, E/A, septal E/e’, lateral E/e’, average E/e’, left ventricular ejection fraction (LVEF) and diaphragm thickening fraction (DTF) 30 min after starting the SBT. The sensitivities, specificities, positive predictive values, and negative predictive values of the parameters were calculated. The best diagnostic threshold for each index was defined as the value providing the greatest Youden index (sensitivity + specificity − 1). Patients were grouped according to cutoff values derived from ROC curve analysis, and potential independent variables affecting the weaning outcome were then identified by univariate regression analysis. All parameters with a *p* value ≤ 0.1 in univariate analysis were included in a multivariable binary logistic regression model to identify independent factors of weaning failure after adjusting for potential confounding factors such as age, sex, and APACHE II score. A two-tailed *p* value ≤ 0.05 was considered statistically significant. All data were analyzed using R version 4.0.2.

## Results

**Fig. 2:**
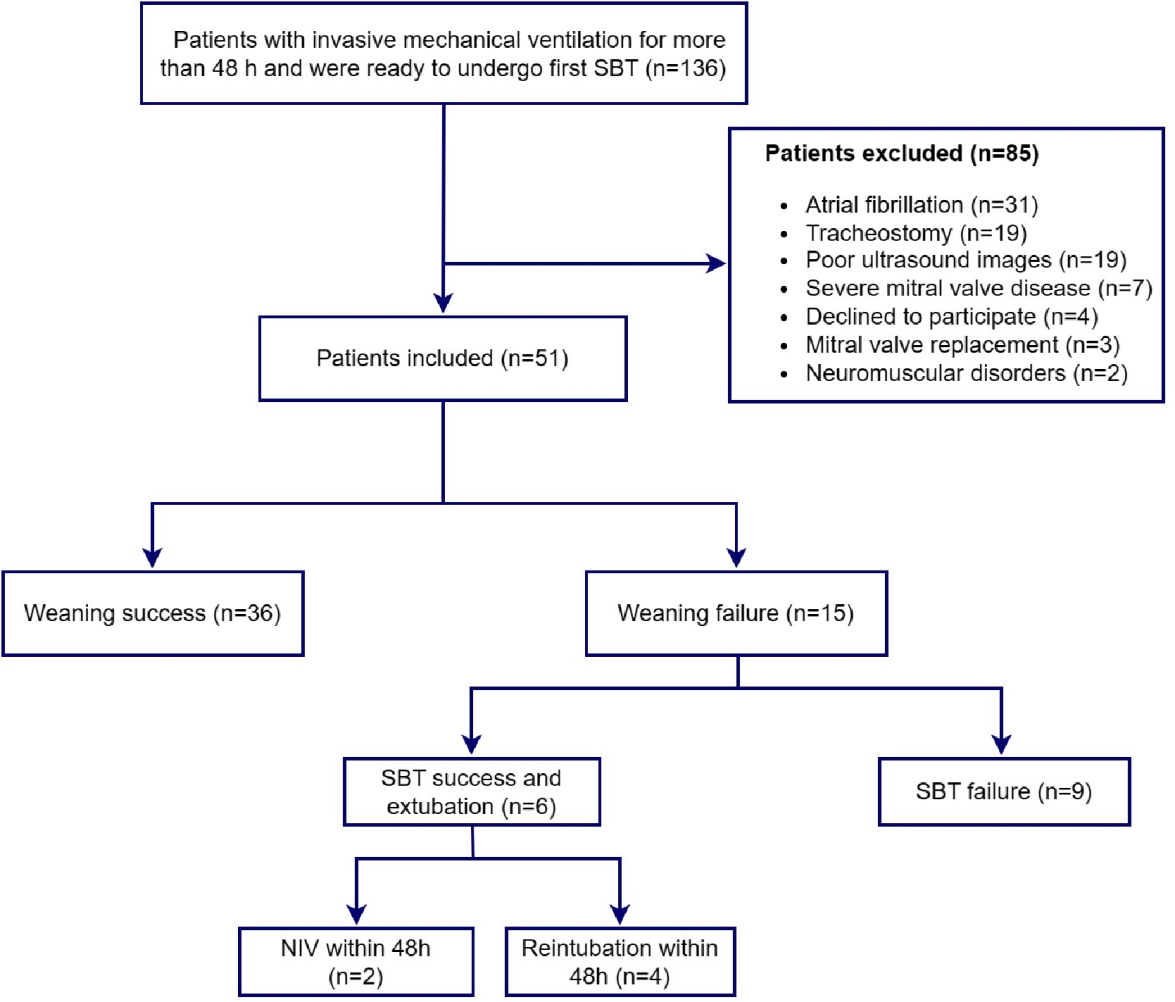
Study flowchart. *SBT* spontaneous breathing trial; *NIV* non-invasive ventilation

During the study period, a total of 136 patients received invasive MV for > 48 h and were ready to undergo their first SBT. Fifty-one patients were finally included in the study (38 males; median age 72 years (55–82); APACHE II: 19 (16–22); SOFA: 6 (4–8)). At the time of inclusion, the median duration of MV was 6 (3–8) days. Of the 51 patients, 15 (29.4%) failed weaning, including nine (17.6%) who failed SBT and six (11.8%) who required non-invasive or invasive ventilation within 48 h of extubation (4 reintubated, 2 received non-invasive ventilation). A flowchart of the study is shown in Fig. 2. The demographic characteristics, laboratory findings, cumulative fluid balance over the last 3 days, ventilator parameters prior to SBT, length of ICU stay, and ICU mortality did not differ significantly between the weaning success and failure groups (Table 1).

**Table 1:** Patient characteristics

	All patients (*n* = 51)	Weaning success (*n* = 36)	Weaning failure (*n* = 15)	p value
Males, n (%)	38 (75)	29 (81)	9 (60)	0.125
Age, years	72 (55–82)	72 (59–81)	77 (56–82)	0.612
**Comorbidities, n (%)**				
Hypertension	16 (31)	10 (28)	6 (40)	0.599
Diabetes mellitus	13 (25)	8 (22)	5 (33)	0.633
Ischemic cardiomyopathy	8 (16)	6 (17)	2 (13)	0.901
COPD	4 (8)	2 (6)	2 (13)	0.712
Chronic renal failure	5 (10)	5 (14)	0 (0)	0.316
Dilated cardiomyopathy	2 (4)	2 (6)	0 (0)	0.999
**Reason for ICU admission, n (%)**				
Pneumonia	23 (45)	15 (42)	8 (53)	0.446
Neurological	7 (14)	6 (17)	1 (7)	0.618
Cardiac pulmonary oedema	4 (8)	4 (11)	0 (0)	0.439
Cardiac arrest	4 (8)	1 (3)	3 (20)	0.13
Septic shock	3 (6)	1 (3)	2 (13)	0.203
Acute myocardial infarction	2 (4)	2 (6)	0 (0)	0.999
Other	8 (16)	7 (19)	1 (7)	0.471
**Clinical data**				
APACHE II score	19 (16–22)	19 (16–22)	21 (19–21)	0.106
SOFA score	6 (4–8)	6 (5–8)	6 (5–7)	0.616
Albumin, g/L	32 (29–37)	33 (31–37)	31 (27–33)	0.119
Cumulative fluid balance over last 3 days^*^, mL	550 (-767-1503)	-41 (-847-949)	1300 (375–1746)	0.051
Use of diuretics, n (%)	32 (63)	21 (58)	11 (73)	0.313
Duration of mechanical ventilation, days	6 (3–8)	6 (3–7)	7 (4–9)	0.174
**Mechanical ventilation parameters**				
Pressure-support ventilation, n (%)	46 (90)	31 (86)	15 (100)	0.316
Assist-control ventilation, n (%)	5 (10)	5 (14)	0 (0)	0.316
Pressure-support level, cmH_2_O	13 (11–14)	13 (10–13)	14 (11–15)	0.084
PEEP, cmH_2_O	3 (3–4)	3 (3–4)	4 (3–4)	0.761
FiO_2_, %	40 (35–40)	40 (35–40)	40 (35–40)	0.667
**Clinical outcomes**				
ICU mortality, n (%)	9 (18)	5 (14)	4 (27)	0.275
ICU length of stay, days	12 (6–24)	10 (5–21)	15 (10–24)	0.06

^*^Cumulative fluid balance over the last 2 days in patients with invasive mechanical ventilation < 3 days before the first spontaneous breathing trial (SBT)

*COPD* chronic obstructive pulmonary disease; *ICU* intensive care unit; *APACHE* Acute Physiology and Chronic Health Evaluation; *SOFA* Sequential Organ Failure Assessment; *PEEP* positive end-expiratory pressure

**Fig. 3:**
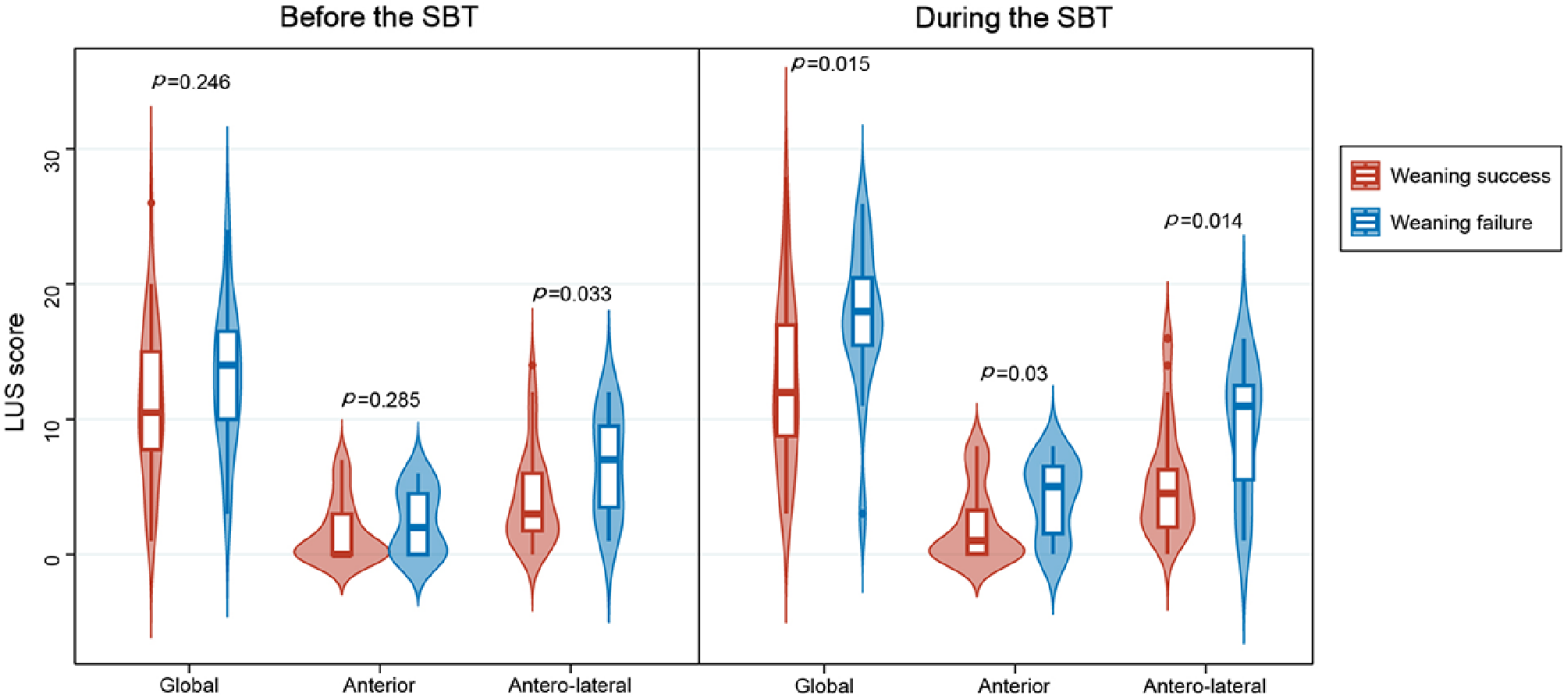
Global, anterior and antero-lateral lung ultrasound (LUS) scores before and during the spontaneous breathing trial (SBT) in patients with successful and failed weaning

There were no significant differences between the groups in terms of clinical, blood gas analysis, and echocardiographic variables before and during the SBT (Electronic Supplementary Material 2 and Table 2). Notably however, the antero-lateral LUS score before the SBT and all LUS scores during the SBT differed significantly between the two groups (Fig. 3), and the DTF was significantly higher in the weaning success group than in the weaning failure group (*p* = 0.048).

**Table 2:** Transthoracic echocardiography and lung and diaphragm ultrasound variables before and during the spontaneous breathing trial

	Before SBT		p value	30 min after starting SBT		p value
	Weaning success (*n* = 36)	Weaning failure (*n* = 15)		Weaning success (*n* = 36)	Weaning failure (*n* = 15)	
**TTE variables**						
LVEF, n (%)			0.624			0.436
30 − 50%	5 (14)	0 (0)		5 (14)	0 (0)	
50 − 70%	28 (78)	15 (100)		29 (81)	15 (100)	
> 70%	3 (8)	0 (0)		2 (6)	0 (0)	
LVOT VTI, cm	21.9 (18.6–25.3)	20.3 (16.1–23.6)	0.247	22.4 (17.6–24.8)	21 (20.3–23.1)	0.598
E, cm/s	83 (63–91)	69 (64–86)	0.368	80 (68–94)	76 (69–82)	0.78
E/A	0.93 (0.78–1.17)	0.78 (0.66–0.95)	0.157	0.83 (0.71–0.95)^*^	0.81 (0.72–1.21)	0.718
e’ septal, cm/s	8 (6–9)	6 (5–8)	0.161	7 (6–9)	7 (6–8)	0.582
E/e’ septal	10.9 (7.3–13.1)	10.3 (9.6–12.3)	0.78	10.8 (8.6–13.3)	10.8 (9.1–12.4)	0.741
e’ lateral, cm/s	10 (8–11)	9 (9–11)	0.917	10 (8–12)	9 (9–10)	0.723
E/e’ lateral	8.1 (6.5–10.1)	7.4 (5.6–9.9)	0.508	8.1 (6.3–10.3)	7.4 (7.2–9.3)	0.975
Average E/e’	9.2 (6.6–11.8)	9.2 (6.7–11.7)	0.893	8.9 (7.2–10.9)	9.0 (7.9–10.1)	0.984
MAPSE, cm	1.4 (1.2–1.5)	1.4 (1.1–1.7)	0.568	1.3 (1-1.7)	1.3 (1.2–1.8)	0.474
TAPSE, cm	2 (1.7–2.3)	1.7 (1.5–2.2)	0.501	2 (1.7–2.3)	1.8 (1.5–2.3)	0.475
RVEDA: LVEDA ratio, n (%)			0.989			0.989
< 0.6	27 (75)	11 (73)		27 (75)	11 (73)	
0.6–1	7 (19)	4 (27)		7 (19)	4 (27)	
> 1	2 (6)	0 (0)		2 (6)	0 (0)	
**LUS variables**						
Global LUS score	11 (8–15)	14 (10–16)	0.246	12 (9–17)^*^	18 (16–20)^*^	0.015
Anterior LUS score	0 (0–3)	2 (0–4)	0.285	1 (0–3)^*^	5 (2–6)^*^	0.03
Antero-lateral LUS score	3 (2–6)	7 (4–9)	0.033	5 (2–6)^*^	11 (7–12)^*^	0.014
Lung consolidation, n (%)	24 (67)	11 (73)	0.64	24 (67)	11 (73)	0.64
**Diaphragm ultrasound variables**						
DE, mm				12 (9–21)	10 (8–14)	0.242
DTF, %				30 (12.4–36.8)	14.7 (12.5–20)	0.048

*SBT* spontaneous breathing trial; *TTE* transthoracic echocardiography; *LVEF* left ventricle ejection fraction; *LVOT* left ventricular outflow tract; *VTI* velocity–time integral; *E* early mitral inflow velocity; *e′* mitral annular early diastolic velocity; *MAPSE* mitral annular plane systolic excursion; *TAPSE* tricuspid annular plane systolic excursion; *RVEDA* right ventricular end-diastolic area; *LVEDA* left ventricular end-diastolic area; *LUS* lung ultrasound; *DE* diaphragm excursion; *DTF* diaphragm thickening fraction

^*^p < 0.05 30 min after starting SBT vs. before SBT

**Fig. 4:**
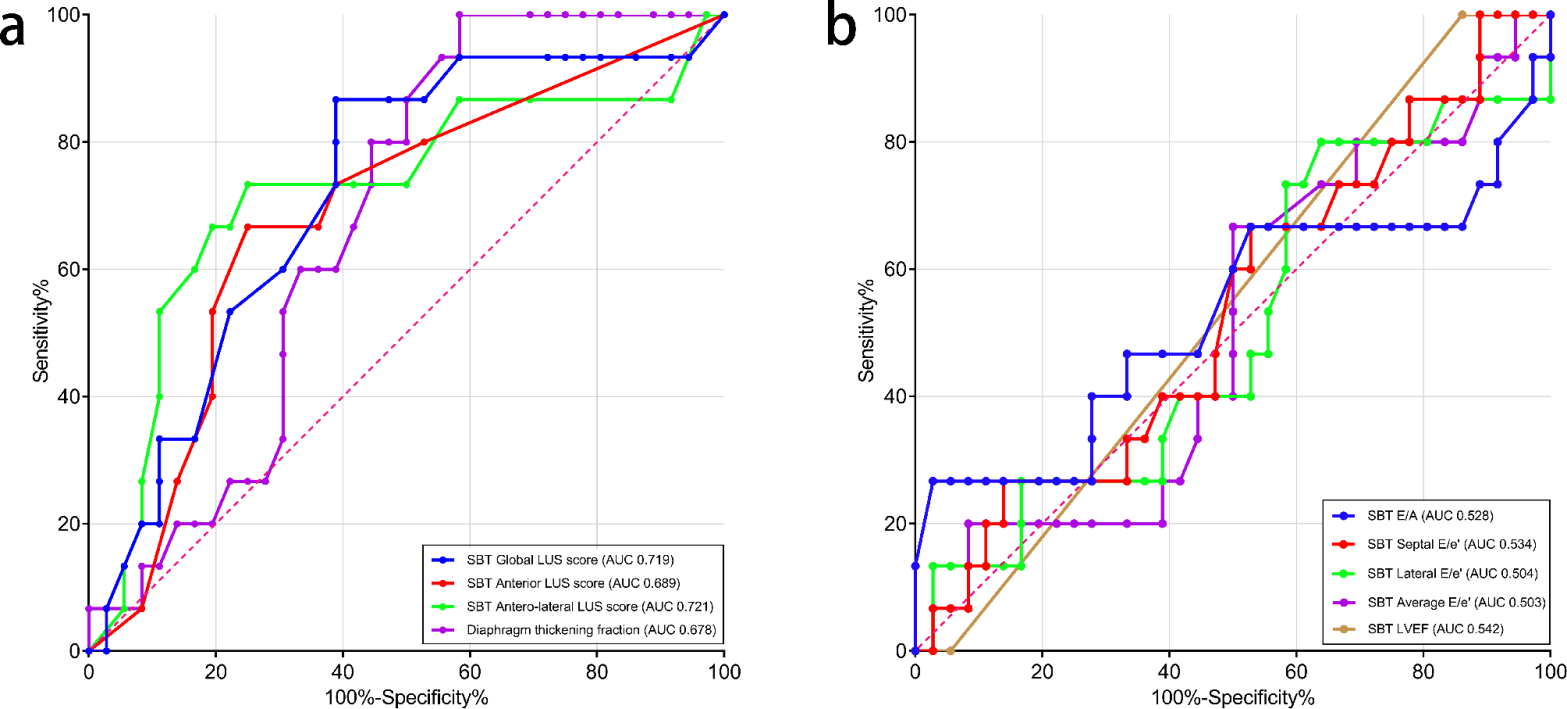
Receiver operating characteristic curves for lung ultrasound (LUS) scores and diaphragm ultrasound **(a)** and echocardiography **(b)** parameters during the spontaneous breathing trial (SBT) to predict weaning failure. *AUC* area under the curve

ROC curve analysis was conducted to assess the diagnostic accuracy of ultrasound variables for predicting weaning failure (Fig. 4). The best cutoff value and diagnostic performance for each variable are shown in Table 3. There was no correlation between any of the LUS scores and average E/e′ during the SBT.

**Table 3:** Accuracy of echocardiography and lung and diaphragm ultrasound variables during the SBT for predicting weaning failure

Variables	Threshold	AUC (95% CI)	p value	Sensitivity (%)	Specificity (%)	PPV (%)	NPV (%)
SBT Global LUS score	14	0.719 (0.564–0.875)	0.014	86.7	61.1	48.1	91.7
SBT Anterior LUS score	4	0.689 (0.529–0.849)	0.035	66.7	75	52.6	84.4
SBT Antero-lateral LUS score	7	0.721 (0.545–0.897)	0.014	73.3	75	55	87.1
DTF	31	0.678 (0.532–0.823)	0.047	100	41.7	41.7	100
SBT E/A	0.54	0.528 (0.322–0.733)	0.757	26.7	97.2	80	76.1
SBT Septal E/e’	10.3	0.534 (0.358–0.711)	0.702	66.7	47.2	34.5	77.3
SBT Lateral E/e’	6.9	0.504 (0.321–0.687)	0.967	80	36.1	34.3	81.2
SBT Average E/e’	8.8	0.503 (0.326–0.68)	0.975	66.7	50	35.7	78.3
SBT LVEF	50	0.542 (0.379–0.705)	0.642	100	13.9	32.6	100

*AUC* area under the curve; *CI* confidence interval; *PPV* positive predictive value; *NPV* negative predictive value; *SBT* spontaneous breathing trial; *LUS* lung ultrasound; *DTF* diaphragm thickening fraction; *E* early mitral inflow velocity; *A* late mitral inflow velocity; *e´* mitral annular early diastolic velocity; *LVEF* left ventricle ejection fraction

Multivariate logistic regression analysis identified antero-lateral LUS score > 7 and DTF < 31% during the SBT as independent predictors of weaning failure after adjusting for age, sex, and APACHE II score (Table 4).

**Table 4:** Univariate and multivariate analyses of risk factors for weaning failure

	Univariate analysis		Multivariate analysis	
Variables	OR (95% CI)	*p* value	Adj.^*^ OR (95% CI)	*p* value
Cumulative fluid balance (mL)	1 (1–1)	0.165		
SBT Global LUS score > 14	10.21 (2.36–71.97)	0.005		
SBT Antero-lateral LUS score > 7	8.25 (2.24–36.3)	0.003	19.65 (3.43-211.53)	0.003
Lung consolidation on LUS	1.38 (0.38–5.79)	0.641		
DTF < 31%	10 (1.72-191.12)	0.034	23.96 (2.53-713.02)	0.024

*OR* odds ratio; *CI* confidence interval; *SBT* spontaneous breathing trial; *LUS* lung ultrasound; *DTF* diaphragm thickening fraction

* adjusted for age, sex and APACHE II score

## Discussion

In this prospective study, we conducted a comprehensive ultrasound assessment of the heart, lungs, and diaphragm in a general ICU population before and during the SBT with low-level pressure support. The main results are as follows: (1) During the SBT, patients who failed to wean had higher LUS scores and lower DTF, as compared to patients who were successfully weaned. (2) No significant difference was found between patients who were successfully weaned and those who failed in echocardiographic parameters either before or during the SBT. (3) LUS scores and DTF demonstrate moderate diagnostic performance in predicting weaning failure, and an antero-lateral LUS score > 7 and DTF < 31% during the SBT were independent predictors of weaning failure.

The weaning process induces significant physiological changes in multiple organs, which can result in decreased aeration of the pulmonary parenchyma, such as alveolar decruitment, pulmonary edema, and atelectasis, ultimately leading to weaning failure [4, 8, 13, 14]. LUS has emerged as a valuable technique for assessing lung aeration in critically ill patients due to its accuracy, ease of use, and noninvasive nature [15, 16], and thus offers several advantages in detecting lung issues during the weaning process. Bouhemad et al. recently conducted a prospective observational study involving 40 elderly patients at high risk of weaning or extubation failure and found that patients who experienced weaning or extubation failure had significantly higher LUS scores at the end of the SBT. Both global and antero-lateral LUS scores at the end of the SBT were identified as independent risk factors for weaning or extubation failure [17]. Our study yielded similar findings in a general population of critically ill patients at their first attempt to wean from MV. These findings highlight the utility of LUS as a bedside tool for identifying patients at risk of weaning failure and accurately predicting weaning outcomes.

The transition from invasive MV to spontaneous breathing is associated with increased cardiac preload and afterload, potentially triggering cardiogenic pulmonary edema in cases of volume overload or left ventricular (LV) systolic or diastolic dysfunction [18, 19]. Echocardiography is a useful tool for assessing cardiac performance during the weaning process [20, 21]. Several studies have examined the value of echocardiographic parameters for predicting weaning outcomes [22–25]. A recent meta-analysis highlighted the pivotal roles of LV diastolic function dysfunction (E/e′, e′ wave, E wave) and increased LV filling pressure (E/e′) in weaning failure, while the influence of LV systolic dysfunction remains ambiguous [5]. In contrast to previous studies, the present results showed that echocardiographic parameters of diastolic function were not associated with weaning failure. A plausible explanation might be that the effects of therapeutic interventions. Up to 63% of our patients had received diuretics before SBT, while the reduction in cardiac preload induced by diuretics may lead to soften the effects of SBT on LV filling pressure [26, 27]. This postulate was more or less supported by the fact that the E/e′, as a routine indicator of LV filling pressure, was not significantly increased during the SBT. Such findings indicate that patients may adapt better to SBT-induced hemodynamic changes after diuretic therapy and a more negative fluid balance. Similarly, no significant difference in LVEF was found between the weaning success and weaning failure groups. This could be attributed to the relatively low incidence of impaired LV systolic function in our cohort. Only 10% of our patients had an LVEF lower than 50%.

WIPO is a common cause of weaning failure. LUS is considered to be an accurate and useful diagnostic tool for detecting WIPO [6]. B-lines detected by LUS are strongly correlated with increased extravascular lung water [28, 29], while multiple B-lines in bilateral lung regions are considered to be characteristic of cardiogenic pulmonary edema [30]. Ferre et al. recently found that an increase in the number of B-lines ≥ 6 on four anterior thoracic quadrants during SBT allowed the diagnosis of WIPO with the best accuracy [6]. Nevertheless, our study found no correlation between LUS scores and LV filling pressure (E/e′), suggesting that WIPO might not be the main mechanism of lung aeration loss in our study population. There are many possible causes of lung aeration loss during weaning, including alveolar decruitment secondary to loss of positive end-expiratory pressure, diaphragm dysfunction, atelectasis, and pulmonary edema. An elevated LUS score does not necessarily equate to a rise in LV filling pressure and cardiogenic pulmonary edema. Similar results were obtained in a previous study involving an elderly population [17]. The complex pathophysiological changes that affect lung aeration during weaning highlight the need for an integrative ultrasound assessment.

The diaphragm acts as the primary inspiratory muscle and plays a crucial role in ventilation. The existing evidence suggests that 44% of patients experience a decrease in diaphragm thickness in the first 72 h of MV, and this atrophy was associated with low diaphragm contractile activity [31]. The diaphragm atrophy may contribute to weaning failure and prolongation of invasive mechanical ventilation [32]. Therefore, assessing diaphragm function could help predict the capacity of the respiratory muscles to cope with the increased respiratory load. However, in the present study, DTF is a more accurate parameter of diaphragm contractile activity than DE during a low-level PSV. Actually, during assisted ventilation, DE is the result of the combined forces of active diaphragm contraction and passive displacement caused by the pressure from the ventilator [33]. In this case, there is no means to distinguish which part of displacement is passive, and which is active. Moreover, DE varies depending on the patient’s position, breathing pattern and abdominal and/or thoracic pressure [34]. Consequently, DE may not be a reliable parameter for monitoring diaphragm contractile activity during PSV. In contrast, DTF is primarily influenced by active diaphragm contraction rather than passive insufflation during PSV [35]. Umbrello et al. demonstrated a significant correlation between DTF and classic parameters of inspiratory muscle effort, such as esophageal pressure-time product per breath (PTPes) and diaphragmatic pressure-time product per breath (PTPdi) [36], indicating that DTF serves as a dependable indicator of respiratory effort in PSV.

One of the strengths of our study was the comprehensive bedside ultrasound assessment of cardiac, lungs, and diaphragm functions with multiple parameters. Weaning from MV is a complex process, involving pathophysiological changes in multiple organs as well as cross-talk between organs [13]; assessing a single organ may thus overlook potential risk factors for weaning failure. Moreover, dynamic ultrasound assessments were conducted both before and during the SBT. An integrative, dynamic, and comprehensive ultrasound assessment of the heart, lungs, and diaphragm not only enables clinicians to evaluate the risk of weaning failure more accurately, but also helps identify underlying mechanisms and guide further treatments in patients with weaning failure.

Our study also had some limitations. First, the sample size was small. We only enrolled patients from two centers and only included patients undergoing their first SBT. This may limit the generalizability of our findings. Second, SBT was performed with low-level pressure support, which is not as challenging for the heart as T-tube. The current results may thus not translate to patients undergoing SBT with T-tube. Third, our study did not classify diastolic dysfunction according to the American Society of Echocardiography (ASE) and European Association of Cardiovascular Imaging (EACVI) Recommendations [37]. Since the E/e’ is accepted as a surrogate parameter of LV filling pressure, we used E/e’ as an index of diastolic function in this study. Fourth, ultrasound examination is operator-dependent. Inter- and intra-operator reproducibility were not assessed. However, in the present study, all ultrasound examinations were performed by experienced physicians with advanced-level training and confirmed off-line by an independent operator, which may reduce the intra- and inter-operator variability to some extent. Finally, ultrasound examination may be difficult in cases with a poor viewing window due to obesity, subcutaneous emphysema, and/or thoracic dressings.

## Conclusion

In conclusion, the current study showed that LUS and diaphragm ultrasound can help to predict weaning failure in critically ill patients undergoing an SBT with low-level pressure support. Among the ultrasound parameters, an antero-lateral LUS score > 7 and DTF < 31% during the SBT were independent predictors of weaning failure. Further studies should focus on optimizing the protocol of combined cardiac, lung, and diaphragm ultrasound assessment. In addition, interventional studies are required to validate the effectiveness of an ultrasound-guided strategy for MV weaning in a broader population.

### Electronic supplementary material

Below is the link to the electronic supplementary material.

**Supplementary Material 1:** The eligibility criteria for the spontaneous breathing trial (SBT) and the acquisition of images and measurement of parameters for transthoracic echocardiography (TTE), lung ultrasound (LUS), and diaphragm ultrasound.

**Supplementary Material 2: Table S1.** Clinical and blood gas analysis variables before and during the spontaneous breathing trial (SBT).

## Data Availability

The datasets used and/or analyzed during the current study are available from the corresponding author on reasonable request.

## Abbreviations

MV: mechanical ventilation
LUS: lung ultrasound
TTE: transthoracic echocardiography
SBT: spontaneous breathing trial
DTF: diaphragm thickening fraction
WIPO: weaning-induced pulmonary edema
ICU: intensive care units
APACHE: Acute Physiology and Chronic Health Evaluation
SOFA: Sequential Organ Failure Assessment
ROC: receiver operating characteristic
DE: diaphragm excursion
LVEF: left ventricular ejection fraction
