# Supplementary material for: Combined cardiac, lung, and diaphragm ultrasound for predicting weaning failure during spontaneous breathing trial
Source: Ann Intensive Care. 2024 Apr 20;14:60. doi: 10.1186/s13613-024-01294-2 (PMC11031537; doi:10.1186/s13613-024-01294-2)
Supplement: Supplementary file 1 — Supplementary Material 1: The eligibility criteria for the spontaneous breathing trial (SBT) and the acquisition of images and measurement of parameters for transthoracic echocardiography (TTE), lung ultrasound (LUS), and diaphragm ultrasound. [file 13613_2024_1294_MOESM1_ESM.pdf]

Additional file 1

Combined cardiac, lung, and diaphragm ultrasound for predicting weaning failure during spontaneous breathing trial

Patients

Patients were eligible for a spontaneous breathing trial (SBT) if they fulfilled the following criteria: (1) resolution or improvement of disease leading to mechanical ventilation; (2) adequate oxygenation (arterial oxygen saturation > 90% with inspired oxygen fraction ≤ 0.4, or arterial oxygen partial pressure to inspired oxygen fraction ≥ 150 mmHg, both with positive end-expiratory pressure ≤ 8 cmH_2O); (3) adequate pulmonary function (respiratory frequency ≤ 35 breaths/min, tidal volume ≥ 5 mL/kg ideal body weight and no significant respiratory acidosis); (4) stable cardiovascular status (heart rate ≤ 140 beats/min, systolic blood pressure 90–160 mmHg without or with minimal vasopressors); (5) stable metabolic status; (6) adequate mentation; (7) absence of excessive tracheobronchial secretion; and (8) effective cough reflex.

Ultrasound examination

Transthoracic echocardiography (TTE)

TTE was performed using a S5-1 or 3Sc phased array (1–5 MHz) probe.

Left ventricular (LV) ejection fraction was assessed by visual evaluation from apical four-chamber view, and divided into four grades (< 30%; 30%–50%; 50%–70%; > 70%). The mitral annular plane systolic excursion was measured with M-mode imaging for longitudinal systolic function assessment. We measured the velocity time integral of the LV outflow tract from the apical five-chamber view using pulsed wave Doppler.

LV diastolic function was assessed by pulsed wave Doppler and tissue Doppler imaging from apical four-chamber view. Mitral inflow velocities were evaluated with a 1- to 2-mm sample volume placed at the mitral valve tip by pulsed wave. Peak flow velocities were measured during early diastole (E wave) and atrial systole (A wave) and the E/A ratio was computed. Pulsed-wave tissue Doppler imaging was performed with a 5-mm sample volume placed at the junction of the septal and lateral mitral valve annulus. The early (e′) diastolic velocity and late (a′) diastolic velocity at the septal and lateral mitral valve annulus were recorded. The septal, lateral, and average E/e′ ratio (2 × E / [septal e′+ lateral e′]) were computed, respectively.

Right ventricular (RV) function was assessed qualitatively by the RV:LV area ratio at the end of ventricular diastole, and classified into three grades, as described elsewhere [1]: normal size (right ventricular end-diastolic area [RVEDA] / left ventricular end-diastolic area [LVEDA] < 0.6), moderately enlarged (RVEDA / LVEDA > 0.6), or severely enlarged

(RVEDA / LVEDA > 1). RV global systolic function was assessed by measuring the M-mode-derived tricuspid annular plane systolic excursion from RV-focused apical four-chamber view.

Lung ultrasound (LUS)

LUS was performed using a C5-1 or C1-5 convex array (1–5 MHz) probe. A comprehensive scan was taken in six regions for each hemithorax: superior and inferior areas in the anterior, lateral, and posterior regions using anterior and posterior axillary lines as landmarks. Each region was scored according to the LUS aeration pattern as follows [2]: (1) normal aeration (score 0), presence of lung sliding with A lines or fewer than two isolated B lines; (2) moderate loss of lung aeration (score 1), presence of three or more well-defined B lines in a single intercostal space; (3) severe loss of lung aeration (score 2), multiple coalescent B lines; and (4) lung consolidation (score 3), presence of a tissue-like pattern. Points were allocated for a given region of interest according to the worst ultrasound pattern observed. Global, anterior, and antero-lateral LUS scores were calculated as the sum of all 12 regions, anterior four regions, and antero-lateral eight regions, ranging from 0–36, 0–12, and 0–24, respectively. The LUS examination protocol and scoring system are shown in Fig. S1.

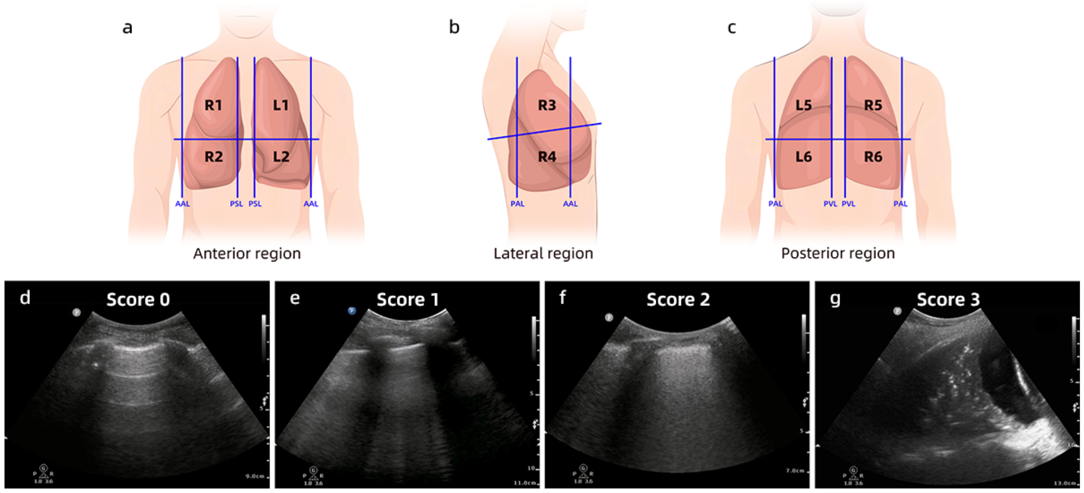

Fig. S1 Lung ultrasound (LUS) examination protocol and scoring system. Each hemithorax was separated into six regions: anterior **(a)**, lateral **(b)**, and posterior **(c)** regions were separated by the anterior and posterior axillary lines, with each region divided into upper and lower portions. Examples of LUS findings according to the scoring system: **(d)** normal lung aeration, scored as 0; **(e)** moderate loss of lung aeration, scored as 1; **(f)** severe loss of lung aeration, scored as 2; and **(g)** lung consolidation, scored as 3. *PSL* parasternal line; *AAL* anterior axillary line; *PAL* posterior axillary line; *PVL* paravertebral line

Diaphragm ultrasound

Diaphragm excursion (DE) was measured with a C5-1 or C1-5 convex array (1–5 MHz) probe. Only right hemidiaphragm measurements were performed. Using the liver as an acoustic window, the probe was placed immediately below the right costal margin on the anterior axillary or midclavicular line. B-mode was initially used to obtain optimal ultrasound

imaging of the diaphragm and to select the exploration line. The M-mode selected line was adjusted to be perpendicular to the movement of the hemidiaphragm. The diaphragm appeared as a single thick echogenic line in M-mode view. DE was measured as the vertical distance from the end of inspiration to the end of expiration [3].

The diaphragm was visualized as reported previously [4], by placing the linear array probe (5–12 MHz) perpendicular to the chest wall in the eighth or ninth intercostal space, between the anterior axillary and mid-axillary lines, to observe the zone of apposition of the muscle 0.5–2 cm below the costophrenic sinus. In this area, the diaphragm was visible as a structure comprising three distinct layers in B-mode: a non-echogenic central layer (muscle) bordered by two echogenic layers (pleural line and peritoneal line). To obtain adequate diaphragmatic images in M-mode, the diaphragm thickness (DT) was measured as the muscle layer at both end-inspiration and end-expiration phases. Measurements of DE and DT are shown in Fig. S2. The DTF was calculated from the following formula: thickness at end-inspiration − thickness at end-expiration)/thickness at end-expiration × 100%.

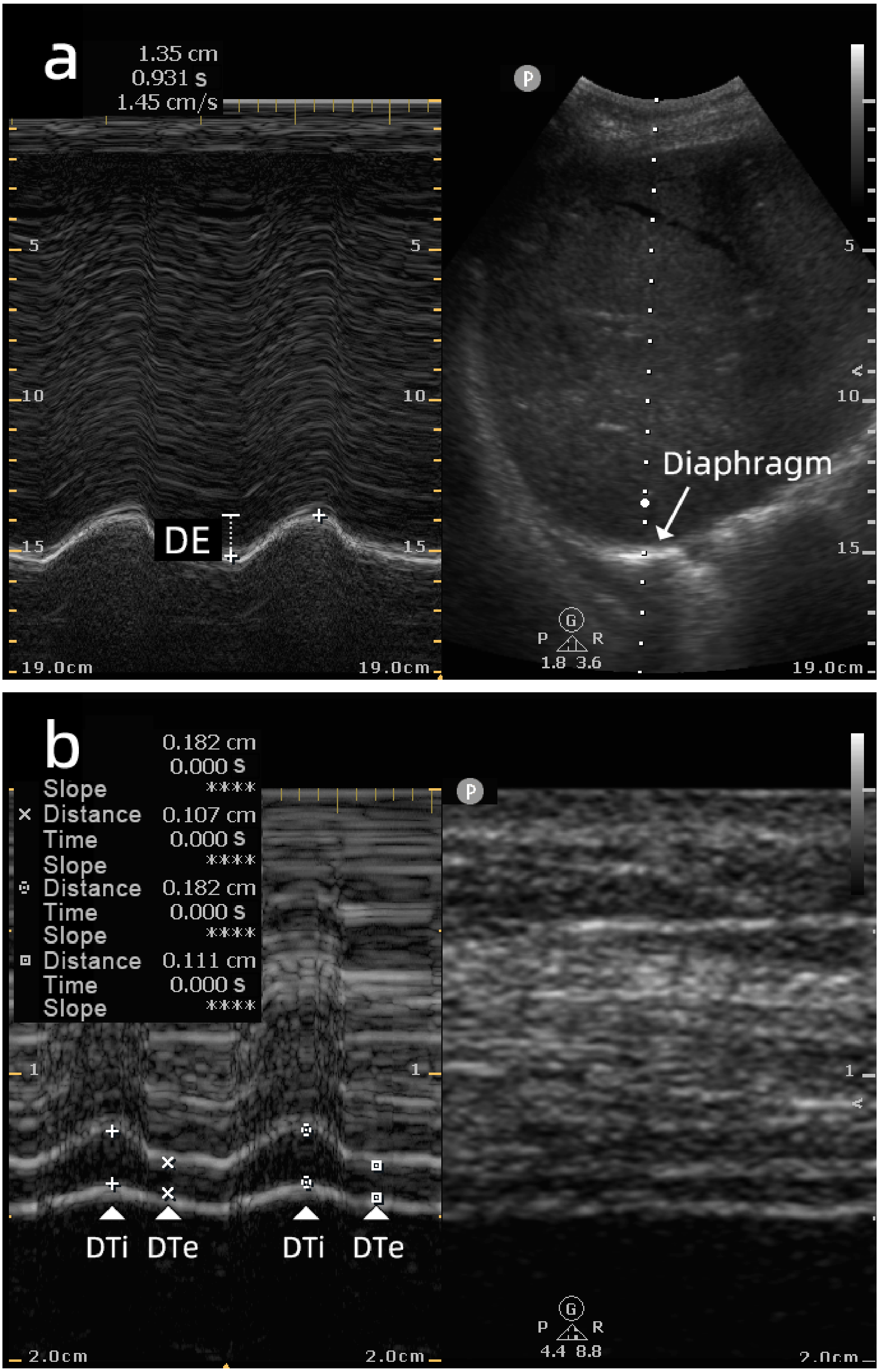

Fig. S2 Measurement of diaphragm excursion **(a)** and diaphragm

thickness **(b)**. *DE* diaphragm excursion; *DTi* diaphragm thickness at end-inspiration; *DTe* diaphragm thickness at end-expiration

References

1. Rudski LG, Lai WW, Afilalo J, Hua L, Handschumacher MD, Chandrasekaran K, et al. Guidelines for the Echocardiographic Assessment of the Right Heart in Adults: A Report from the American Society of Echocardiography. Journal of the American Society of Echocardiography. 2010;23:685–713.

2. Mongodi S, De Luca D, Colombo A, Stella A, Santangelo E, Corradi F, et al. Quantitative Lung Ultrasound: Technical Aspects and Clinical Applications. Anesthesiology. 2021;134:949–65.

3. Matamis D, Soilemezi E, Tsagourias M, Akoumianaki E, Dimassi S, Boroli F, et al. Sonographic evaluation of the diaphragm in critically ill patients. Technique and clinical applications. Intensive Care Med. 2013;39:801–10.

4. Tuinman PR, Jonkman AH, Dres M, Shi Z-H, Goligher EC, Goffi A, et al. Respiratory muscle ultrasonography: methodology, basic and advanced principles and clinical applications in ICU and ED patients—a narrative review. Intensive Care Med. 2020;46:594–605.
