# Supplementary material for: Combined cardiac, lung, and diaphragm ultrasound for predicting weaning failure during spontaneous breathing trial
Source: Ann Intensive Care. 2024 Apr 20;14:60. doi: 10.1186/s13613-024-01294-2 (PMC11031537; doi:10.1186/s13613-024-01294-2)
Supplement: Supplementary file 2 — Supplementary Material 2: Table S1. Clinical and blood gas analysis variables before and during the spontaneous breathing trial (SBT). [file 13613_2024_1294_MOESM2_ESM.pdf]

Additional file 2

Table S1 Clinical and blood gas analysis variables before and during the spontaneous breathing trial

	**Before SBT**		p value	**30 min after starting SBT**		p value
	Weaning success (n=36)	Weaning failure (n=15)		Weaning success (n=36)	Weaning failure (n=15)	
**Clinical variables**						
Heat rate, beats/min	92 (79-98)	95 (84-104)	0.475	88 (79-101)	98 (91-101)	0.396
Systolic blood pressure, mmHg	133 (121-143)	132 (119-141)	0.664	138 (119-149)	149 (132-155)	0.104
Diastolic blood pressure, mmHg	69 (61-74)	64 (59-73)	0.656	65 (57-75)	66 (60-83)	0.426
Mean arterial pressure, mmHg	90 (80-97)	88 (77-98)	0.918	87 (80-94)	94 (90-98)*	0.086
Respiratory rate, beats/min	16 (15-19)	14 (13-18)	0.191	19 (18-23)*	19 (17-24)*	0.917
SpO_2, %	100 (99-100)	100 (99-100)	0.68	100 (100-100)	100 (99-100)	0.134
Tidal volume, mL	474 (422-548)	452 (409-497)	0.62	496 (433-573)	462 (418-489)	0.301
**Blood gas analysis variables**						
pH	7.44 (7.4-7.46)	7.44 (7.41-7.46)	0.901	7.43 (7.4-7.46)	7.41 (7.4-7.45)	0.62
PaO_2, mmHg	129 (106-150)	133 (115-141)	0.78	130 (108-158)	98 (85-142)	0.109
$PaCO_2$, mmHg	40 (35-43)	44 (36-49)	0.215	38 (36-43)	45 (33-50)	0.341
HCO_3^-, mmol/L	26.1 (23.6-29)	29.1 (24.9-30.5)	0.247	25 (23.6-28.5)	28.2 (24.4-32.9)	0.137
Lactate, mmol/L	1.4 (1.2-1.8)	1.5 (1.4-1.6)	0.347	1.5 (1-1.7)	1.5 (1.3-1.9)	0.172

SBT spontaneous breathing trial; *SpO_2* pulsed oxygen saturation

$^*p < 0.05$ 30 min after starting SBT vs. before SBT
